# Patients, caregivers and health‐care professionals’ experience with an interdisciplinary intervention for people with multimorbidity in primary care: A qualitative study

**DOI:** 10.1111/hex.13035

**Published:** 2020-02-08

**Authors:** Patrice Alain Ngangue, Catherine Forgues, Tu Nguyen, Maxime Sasseville, Frances Gallagher, Christine Loignon, Moira Stewart, Judith Belle Brown, Maud‐Christine Chouinard, Martin Fortin

**Affiliations:** ^1^ Faculty of Medicine and Health Sciences Université de Sherbrooke Sherbrooke QC Canada; ^2^ Westmead Applied Research Centre The University of Sydney Camperdown NSW Australia; ^3^ Department of health sciences Université du Québec à Chicoutimi Chicoutimi Québec Canada; ^4^ Centre for Studies in Family Medicine Department of Family Medicine Schulich School of Medicine & Dentistry Western University London Canada

**Keywords:** chronic disease, multimorbidity, patient care team, patient‐centred care, primary health care, qualitative research, self‐management

## Abstract

**Background:**

Multimorbidity challenges the health‐care system and requires innovative approaches. In 2015, a 4‐month patient‐centred interdisciplinary pragmatic intervention was implemented in primary care with the aim of supporting self‐management for patients with multimorbidity.

**Objective:**

To explore the perceptions and experiences of health‐care professionals, patients and their caregivers with a 4‐month patient‐centred interdisciplinary pragmatic intervention in primary care.

**Design:**

A descriptive, qualitative study using semi‐structured interviews was conducted.

**Setting and participants:**

A purposive sample of 30 participants was recruited from seven family medicine groups including patients, caregivers and health‐care professionals (HCPs). Interviews were analysed using Thorne's interpretive description approach.

**Results:**

Findings were grouped into the benefits and challenges of participating in the intervention. The programme allowed patients to adopt realistic and adapted objectives; to customize interventions to the patient's reality; and to help patients gain confidence, improve their knowledge, skills and motivation to manage their condition. Interprofessional collaboration eased the exchange of information via team meetings and electronic medical records. Challenges were related to collaboration, communication, coordination of work and integration of newly relocated HCPs mainly due to part‐time assignments and staff turnover. HCPs part‐time schedules limited their availability and hindered patients’ follow‐up.

**Discussion and conclusion:**

This intervention was useful and rewarding from the HCPs, patients and caregivers’ perspective. However, to ensure the success of this complex interdisciplinary intervention, implementers and managers should anticipate organizational barriers such as availability and time management of relocated HCPs.

## 1 INTRODUCTION

With the ageing of the population, the prevalence and morbidity of chronic diseases (CD) will continue to grow.^1^ People who suffer from multiple CD (multimorbidity) are particularly vulnerable.^2^ The complex management of this population can lead to inappropriate use of health services, resulting in fragmented and less efficient care and higher costs for health systems.^3^ This situation is a challenge for health‐care systems to ensure accessibility, quality and continuity of care.^4^ There is a broad international consensus that multimorbidity is very common and well addressed in primary care settings.^5^, ^6^ Like other countries facing similar health issues, Canada is committed to health system reform focusing on strengthening primary health care, interprofessional collaboration and integrated clinical care.^7^ In the province of Quebec, this reform resulted in the creation of a CD prevention and management primary care framework based on the chronic care model.^8^, ^9^ The cornerstone of this approach is the integration of prevention and management of CD services by health‐care professionals providing interdisciplinary care in primary care.^9^ The adoption by health‐care professionals of the patient‐centred care (PCC) approach when meeting with the patient with a chronic illness is essential.^10^ The PCC approach makes it possible to provide patients with self‐management options adapted to their preferences.^10^, ^11^ Management of patients with chronic diseases is best addressed by an interdisciplinary team in a climate of professional collaboration. The interprofessional collaboration allows health‐care professionals to share their expertise and point of view to formulate common goals aiming to improve or maintain the patient's health status.^12^, ^13^ In Quebec, this approach has been implemented and evaluated with positive health effects like improvement in self‐management, physical activity levels and increased consumption of fruits and vegetables.^14^ A growing number of qualitative studies have explored the experience of patients with multimorbidity.^15^, ^16^, ^17^, ^18^ There have also been numerous studies of the experiences and challenges of health‐care providers of patients with multimorbidity.^19^, ^20^, ^21^ These articles were mainly interested in the experiences of people aged 65 and over, suffering from multiple chronic diseases, followed in primary care (usual care). Only a few studies have emphasized the combined experience of multimorbidity from the perspectives of patients, family caregivers and health‐care professionals. 22, 23, 24 Moreover, no study has looked at the experiences of patients with multimorbidity, their caregivers and health professionals as part of an integrated, interdisciplinary intervention in primary care.

Hence, this study aimed to explore the perceptions and experiences of health‐care professionals, patients and their caregivers with a four‐month interdisciplinary intervention to support self‐management of patients with multimorbidity.

## 2 METHODS

### 2.1 Patient‐centred care (PCC) approach

A four‐month, pragmatic, interdisciplinary intervention for the prevention and management of chronic diseases aimed at supporting self‐management of patients with multimorbidity in primary care was conducted from April 2016 to July 2017. Patients were assessed for eligibility by family physicians or registered nurses. Each eligible patient was provided with a one‐hour initial assessment by a primary care nurse to create an intervention plan focused on their needs and according to their objectives. Patients were directed to other health‐care professionals (nutritionists, kinesiologists or the respiratory therapist) according to their intervention plan. Each patient's intervention had to be based on the educational and coaching content of the training (patient‐centred care approach for patients with multimorbidity, self‐management support and motivational interviewing). Professionals had an average of 7.8 hours of training, and patients had an average of 2.6 hours of interprofessional interventions throughout the 4 months of the intervention. Interdisciplinary meetings between family physicians, nurses and other health professionals were to be held to discuss cases and harmonize the intervention plan.

During the implementation phase, health‐care professionals (nutritionists, kinesiologists or respiratory therapists) previously were relocated into family medicine groups (FMGs) and trained on the patient‐centred care (PCC) approach for patients with multimorbidity,^25^, ^26^ interprofessional collaboration,^27^, ^28^ motivational interviewing^29^, ^30^ and self‐management support.^31^, ^32^ Finally, a community practice with key resource persons (nurses, nutritionists, kinesiologists) and a nurse coordinator was created within each FMG. The community of practice aimed to support the integration of the intervention, to ensure the quality of the care, harmonize ongoing changes to practice and consolidate achievements. The intervention logic model and the Template for Intervention Description and Replication (TIDieR) checklist are available in Appendix S2 and S3, respectively.

### 2.2 Study design and research sites

We conducted a descriptive, qualitative study as part of a multijurisdictional (Quebec and Ontario) concurrent triangulation mixed‐methods study described in a previous article.^33^ This approach allowed us to provide a comprehensive description of participants’ experience in plain language while remaining close to the data, minimizing researchers’ influence on interpretation.^34^

Seven of the eleven family medicine groups (FMGs) from Saguenay Lac‐St‐Jean, a region in the province of Quebec, Canada, participated in both aspects of the evaluation (quantitative and qualitative). FMGs are primary care clinics in which family physicians work with other health‐care professionals to provide comprehensive primary care. In rural areas, FMGs may have several sites. As part of this intervention, these other HCPs have been relocated to the physical site of the FMG practice. 35, 36 We followed the consolidated criteria for reporting qualitative studies (COREQ) guidelines for reporting qualitative research (details in Appendix S1).

### 2.3 Participants and sampling strategy

We used purposive sampling strategies including criterion and maximum variation based on age, gender, income, education and FMG location 37, 38 to recruit from the FMGs health‐care professionals (nurses, nutritionists, kinesiologists and a respiratory therapist), patients with multimorbidity followed for at least four months as part of the intervention, and their caregivers who took part in the intervention. To be included, patients had to be cognitively intact, aged 18‐80 years and report three or chronic conditions or risk factors according to the MM21 questionnaire. 39 Every FMG was represented by at least one type of HCP while patients and caregivers came from three FMGs.

Patients were already participants in a pragmatic trial. They were contacted by phone and offered to extend their participation with this optional qualitative study. Family members (caregivers) were recruited by asking patients if it could interest their carer to participate also. A recruitment invitation was sent by email to all health‐care professionals who participated in the programme or its implementation, followed by phone call reminders. Recruitment continued until data saturation was reached, defined as the point at which no new themes emerged. 40

All participants signed a consent form before the interview.

### 2.4 Data collection

Three semi‐structured interview guides tailored to each participant group were developed according to our logic model (see Appendix S2) and pilot tested. The interview guide for health‐care professionals consisted of open‐ended questions related to the health‐care professionals’ expectations and role in the intervention, patients and caregivers’ expectations, barriers and facilitators influencing the efficiency of the intervention, and the impact of the intervention on themselves. For example, health‐care professionals were asked to describe what might facilitate or hinder benefits for patients: ‘In your opinion, what could have facilitated activities of the intervention?’; ‘In your opinion, what could be the barriers that hindered the activities of the program?’ They were also asked their beliefs about the impact of the intervention on the patient: ‘How can the intervention help patients manage their multiple chronic conditions?’ Patients and caregivers were asked about their (or their family member's) experiences and opinions about the intervention: ‘How did the healthcare professionals’ team help you to manage your chronic health problems?’ or ‘How have you appreciate healthcare services offered by the intervention?’

The individual semi‐structured interviews were conducted from October 2016 to September 2017 (six months after the beginning of the intervention) by a research coordinator (TB), a PhD student (MS), a research assistant (BBD) trained in conducting qualitative interviews, and two senior researchers (MCC, MF). They lasted between 23 and 74 minutes (average of 47 minutes) and were conducted face‐to‐face at the various sites. All interviews were audiotaped and took place at a time and place that was convenient for participants. A demographic questionnaire was used to collect information about participants, such as age, gender and professional background. Data collection ended when we had some confidence that the complexity and variation of participant responses were addressing the research question, acknowledging that there is always more to study on the topic.^41^, ^42^

### 2.5 Data analysis

The analysis was guided by Thorne's qualitative methodology on interpretive description (ID).^41^, ^42^ According to Thorne, ID is suited to a: ‘smaller‐scale qualitative investigation of a clinical phenomenon of interest to the discipline to capture themes and patterns within subjective perceptions and generate an interpretive description capable of informing clinical understanding’.^41^, ^42^ This approach also allows for exploring meanings and explanations that may yield application implications. ID studies are focused on clinical realities conducted in naturalistic contexts and are intended to impact clinical care positively. In this way, ID is a suitable approach to guide inquiry into the experience of an interdisciplinary intervention aiming to support self‐management for patients with multimorbidity in primary care from the multiple perspectives of patients, caregivers and health‐care professionals.^41^, ^42^

Audio‐recorded interviews were transcribed verbatim by a trained transcriptionist and cleaned for accuracy by a research assistant. Inductive thematic analysis^29^ of the interviews was used as the analytic approach, which is consistent with the ID design and has been used in other ID studies. Six steps of thematic analysis were followed through the process. A research assistant with qualitative expertise read through all transcripts and identified possible themes (step 1). Two team members developed a coding scheme inductively from the data based on an independent review of three transcripts. Agreement on a final coding scheme was reached by discussions with the research team. Two people independently used this to code all transcripts using NVivo (version.11.0) to assist with data management (step 2). Together, two team members discussed and identified recurring and converging themes across participants. The refined themes were then discussed and agreed upon with other members of the research team (steps 3 and 4). Finally, each theme was named, defined and a written report generated. Key quotes that illustrated each theme were extrapolated from the data (steps 5 and 6).

According to Thorne's suggestions, methodological integrity and rigour were ensured by the following criteria (a) ‘representative credibility’ (data triangulation of sources among the participants); (b) ‘analytic logic’ (accuracy of transcription, memos, audit trail, participants’ phrasing); (c) ‘interpretive authority’ (iterative review of transcripts, several analysts and researchers with unique perspectives).^41^, ^42^

## 3 RESULTS

### 3.1 Characteristics of the sample

Thirty interviews were conducted with HCPs (n = 16), patients (n = 9) and caregivers (n = 5) (Table 1). Only one of the 16 HCPs interviewed was male. HCPs’ ages ranged from 20 to 69 years, and their health‐care experience ranged from three to 24 years. Of the nine patients interviewed, five were men, their age ranged from 47 to 72 years old and the number of years they had been cared for in their FMG ranged from less than one year to 14 years (creation of FMGs). The number of chronic conditions ranged from 3 to 6. The main comorbidities were depression, cardiovascular diseases, diabetes, dyslipidemia and obesity. Every FMG was represented by at least one type of HCP and one to three patients. Finally, the caregivers ranged from 49 to 64 and three caregivers were men. They were all from different FMGs.

**Table 1:** Characteristics of interviewed participants (n = 30)

	n	Gender, (M/F) (n)	Age, range (years)	Health‐care experience, range (years)	FMGs affiliation	
Health‐care professionals (n = 16)					5	
Nurse practitioners	9	0/9	20‐52	12‐24^a^	4	
Nutritionists	4	0/4	20‐69	4‐18^b^	4	
Kinesiologists	2	1/1	20‐30	3‐6	1	
Respiratory therapist	1	0/1	40‐49	13	1	

				Number of years followed in affiliated FMG (years)		Number of chronic conditions
Patients (n = 9)	9	5/4	47‐72^c^	<1‐14	5	3‐6
Family members (n = 5)	5	3/2	49‐64^d^	N/A	5	N/A

Abbreviation: N/A, not applicable.

a Five missing data.

b Two missing data.

c Four missing data.

d Two missing data.

### 3.2 Health‐care professionals, patients and caregivers’ experiences

Experiences related to HCPs’ training, patients’ initial assessment and interdisciplinary interventions have been grouped into (a) the benefits of adopting and using the PCC approach, motivational interviewing and self‐management support; and (b) the benefits and challenges of interdisciplinary collaboration and HCPs’ accessibility.

#### 3.2.1 Benefits to adopt/use the PCC approach

Following their training, HCPs reported effectively using a patient‐centred care approach during their interventions and noted some benefits to this approach. They thought the PCC approach was well adapted to stimulate patients’ investment in their care and could be a promising step towards productive HCP and patient partnership. To do so, HCPs applied different strategies such as adopting realistic and patient‐adapted objectives; adapting their interventions to reflect the patient's reality; and adopting a listening posture.

Firstly, HCPs highlighted the importance of discussions with the patient to set realistic and appropriate goals to preserve their motivation and make positive changes towards their health behaviours. Thus, the adoption of patient‐adapted goals, respecting their pace to make changes and to persist in the paths they have chosen, is perceived as promising for permanent changes as noted here: ‘We often tell patients that a little change, even better than a big change because we gradually develop the lifestyle habit they do not have…with more realistic goals’. (HCP 15: Nurse).

Secondly*,* a majority of HCPs stressed the necessity to adapt their intervention by taking into consideration the vulnerability of some patients, whether with socioeconomic insecurity level or low health literacy. To do so, they favoured customized interventions that reflect the particularity and singularity of each patient*.* One of the patients expressed his appreciation towards HCPs’ efforts to help him understand complex notions and adapt their interventions to his level of comprehension.

I have been met several times to make sure that I understood well. Because it is difficult to understand it [complex notions] completely…These people have been great; they have been very professional…the whole explanation. (Patient 05)

Thirdly, HCPs specified that they preferred a listening and empathic posture to establish and maintain a productive therapeutic relationship. The adoption of a PCC approach appeared to be a way to reinvest in counselling foundations, which focus on the emotional state and care of the patient, especially when the patient was in a vulnerable or emergency state, for example after receiving bad news. Several patients confirmed this point describing how they had appreciated HCPs’ attentive listening and empathy: ‘Well, listening to what I had to say… like eating fruits, it is difficult for me. She [the nutritionist] was listening… she took into account what I said’ (Patient 09).

#### 3.2.2 Benefits of adopting/using motivational interviewing to improve self‐management

Health‐care professionals found that motivational interviewing foundations supported them to help patients gain confidence, knowledge, skills and motivation to manage the physical, social and emotional impact of their disease. HCPs used different strategies: focus on clear objectives and focus on the process.

For instance, many HCPs revealed how they were able to identify, with their patient the objectives they would be interested in and have the motivation to work on. The attention to respect for the patient's wishes and agreement with objectives is often present. They also underlined that follow‐up appointments were essential to maintain the patient's motivation and to stay in action. They emphasized the need to base their practice on observable benefits found during the process to maintain the patient's motivation. Several mentioned that focussing on the observable benefits is the preferred method to maintain the patient's motivation to reach the changes they had targeted.

We have tangible things to explain: Your blood glucose has reduced; also, your triglycerides are better. That gives real stuff… to be able to see we have positive results, and if we do a little bit more, we will have other positive results… (HCP 03: Nutritionist)

Similar findings from the perspective of patients and caregivers were noted. For example, a patient's spouse said: ‘I found it nice she (HCP) was saying: Yes, yes that is great! Keep going on! … I found it great, that was a professional service’. (Caregiver 04).

In addition to motivational interviewing, HCPs’ efforts to ensure patients’ follow‐up, promote knowledge development and educate patients had benefits on patients’ self‐management. Some HCPs found that using self‐management support approach allows them to increase patients’ awareness and mobilization and improve patients’ confidence, self‐esteem and abilities. For example, two nurses have well‐illustrated this by saying: ‘We try to make the patient self‐manage himself. We give them [information], we give them tools, but we want them to take responsibility for their health’. (HCP 07: Nurse). A patient's caregiver expressed well how the programme might have aroused patients’ self‐investment: ‘Now, I find that he takes charge of his health, so that (the interventions) might have made a difference… it is good for sure’ (Caregiver 05).

Furthermore, HCPs noted that the programme had allowed them to contribute to patients’ improvement of confidence, self‐esteem and sense of ability as expressed by one patient:

All these meetings made me see what my health status was. Also, at the same time, I was reassured in the sense that if I had other health problems. I would be able to find care that I need, know where to look for my care… I expect to live longer than I would have believed a few months ago. (Patient 01)

#### 3.2.3 Benefits and challenges of interdisciplinary collaboration

When asked, HCPs expressed some benefits and challenges related to interdisciplinary collaboration, but this was not very prevalent among patients and caregivers.

Collaboration between HCPs during the intervention has resulted in the use of indirect (eg emails and electronic medical records [EMR]) and direct (formal or informal) communication methods. These types of collaboration were found to improve intervention efficacy for HCP and patients. Several HCPs mentioned that using patients’ EMRs to share information made it easier to start a new intervention with the patient. Proximity in the clinics facilitated the communication between the HCPS, especially when they faced with complex problems. A nurse reported that collaboration of HCPs in clinics allowed a better cohesion of the intervention with patients they share, improving the impact of their intervention on patients:

If your doctor always tells you: ‘It would be good if you eat healthier for your diabetes, and I will make you see the nurse’. Then the nurse again tells you the same thing, then the kinesiologists, and the nutritionist… at some point, you get it. (HCP 12: Nurse)

Challenges for interprofessional collaboration were, in most cases, related to the part‐time schedule of newly relocated health‐care professionals, such as nutritionists, kinesiologists and the respiratory therapist than by the nurses. According to many HCPs, this frequent reality induced mostly problems in information exchange as this nurse reported:

Healthcare professionals that we do not often see, who only come half‐day per week, it seems that I see them less often… we are more in contact with those who are regularly there. (HCP 15: Nurse)

Staff turnover also added to this challenge, which might have, according to an HCP, interfered with interdisciplinary team collaboration and coordination:

There has been much movement with nurses during the last two years… for patients; it can be disruptive. If we had nurses that were well integrated with a continuum and were also able to do the link between the different HCPs to make sure there has follow‐ups, coordination, all this’. (HCP 13: Nutritionist)

#### 3.2.4 Challenges of services’ accessibility

Many HCPs expressed reservations on the actual availability of these newly relocated professionals. The main reason expressed by HCPs was their part‐time schedule, which might affect patients' health care and follow‐up due to lack of time to see every patient every time they needed them.

‘This is a problem because I only work a half‐day per week at the clinic. Sometimes, patients do not need to see me during the time I am there. Also, when I am there, I have my schedule, so I may not have time to do something else. Then, the ideal option would be to always be there’. (HCP 17: Respiratory therapist)

A patient also confirmed that missing staff had affected his health care and follow‐up:

‘I expected more appointments with the kinesiologist. I only had one. It took time before I met her too. I know she was on sick leave or something like this, but it ran out of services. I thought I would have had a tighter follow‐up’. (Patient 07)

Furthermore, a caregiver also provides concerns about the services they received during the four‐month programme, mostly about follow‐up, as illustrated by this quote: ‘The only problem I have… is that calls are not answered… I find it very deplorable in the system…’ (Caregiver 02).

In conclusion, accessibility to relocated HCPs was well appreciated by patients, but their limited availability caused by lack of staff and part‐time schedule may have bothered some patients who were expecting more follow‐up.

## 4 DISCUSSION

### 4.1 Summary

This study reveals how the combination of different approaches, including the training of health‐care professionals on patient‐centred care for patients with multimorbidity and self‐management support through motivational interviewing, the relocation of these specialized health‐care professionals and interprofessional collaboration, resulted in meeting and satisfying patients’ specific needs. Moreover, according to the participants, this intervention improved health‐care professionals’ knowledge and practices and lowered the workload by shared tasks, which resulted in a positive HCP experience.

Nevertheless, this study also demonstrates that there is still room for improvement, mostly regarding the needs’ analysis and time management of the relocated health‐care professionals to improve their availability.

### 4.2 Comparison with existing literature

Positive experiences and needs of patients have mainly been addressed by three of the six intervention components: patient‐centred care approach to multimorbidity, health‐care professionals’ relocation into FMGs and interdisciplinary collaboration. These mostly positive experiences are based on concepts underlying the three different approaches used during interventions with patients. The patient‐centred care approach highlights the partnership between patients and health‐care professionals, takes into account patients’ illness experience, context and definition of the goals^10^ and advocates for a paradigm shift in health care, especially about chronic diseases.^43^ This paradigm shift suggests switching to an approach that gives the patient a more active role in the management of their health and daily decision process instead of a more paternalistic approach.^44^, ^45^, ^46^ Considering patients as a partner allows the initiation of a continuous learning process, letting them acquire experiential knowledge about their health, providing them with understandable scientific information and developing technical competencies.^46^, ^47^, ^48^ It also allows patients to assess the quality and adequacy of care regarding their values and preferences.^46^, ^47^, ^48^ Finally, health‐care professionals relied on patients’ knowledge and assessment to adjust their interventions and reach optimal health status.^46^, ^47^, ^48^, ^49^ Thus, these components all contribute to making the patient‐centred care approach so appreciated by patients and health‐care professionals and contributing to patients’ satisfaction. These results are consistent with previous studies,^23^, ^24^ and especially those conducted in French‐speaking regions. For instance, in a recent study conducted in France with the aim to involve patients with chronic conditions in generating ideas for improving their care, most ideas were related to improving physician‐patient discussions, informing patients about their own care, and adapting treatment to patient preferences and context, and also improving the coordination and collaboration in care.^50^ Also, in a qualitative study of patient and family member experience conducted in Quebec and focused on case management in primary care for frequent users of health‐care services with chronic diseases, results showed that patients felt that their needs were taken into consideration, especially regarding access to the health‐care system.^51^

Moreover, health‐care professionals generally integrated the motivational interviewing approach into their patient‐centred care interventions. By allowing health‐care professionals to strengthen patients’ motivation to change and assist in understanding their care,^30^, ^52^ self‐management support promoted by the motivational interviewing approach reinforces and improves the patient‐centred care approach.^31^, ^53^, ^54^ Self‐management support allows HCPs to teach patients how to identify challenges and actively solve problems associated with their illness.^32^ As well, patients have noted high satisfaction with the availability of health‐care professionals due to their relocation in FMGs. For HCPs, proximity and collaboration with new types of health‐care professionals have been particularly appreciated. HCPs view interdisciplinarity as an opportunity to improve communication by holding regular interdisciplinary meetings to increase their mutual understanding of each team member's role, increase the opportunity for shared decision making, and build a joint commitment and team vision encouraging patient‐centred care with the objective to improve health care and satisfaction of patients.^55^ However, relocation of health‐care professionals into FMGs and interdisciplinary collaboration poses a big challenge regarding organization, accessibility and availability of these newly integrated health‐care professionals. Despite these barriers, strengthening of primary care by the integration of a variety of health‐care professionals remains a necessity to manage and follow‐up patients with multimorbidity.^42^, ^43^, ^44^

### 4.3 Implications for research and clinical practice

Results of this study suggest that the intervention is effective in addressing the needs and expectations of patients. Nevertheless, some pitfalls were found in patients’ follow‐up and interprofessional collaboration and should be addressed to ensure better implementation and improve the effectiveness of the intervention. For instance, one should assess the real needs of relocated health‐care professionals in each clinic regarding patient load and work attribution (full or part‐time) and address difficulties encountered in some clinics where patients are moving from one site to the other (FMGs with multiple and/or distant practices). Another barrier that could be addressed would be the problem of communication and knowledge about the role of particular health‐care professionals in an interprofessional team. In our study, this has been observed in some teams where nutritionists and kinesiologists worked. Finally, future interventions should pay close attention to the waiting times to see a health‐care professional. These strategies should consider both patients’ needs and health‐care professionals’ availability. For instance, the findings of a systematic review published in 2017 have suggested that open access scheduling may reduce wait times for primary care appointments.^56^

### 4.4 Strengths and limitations

One of the strengths of this study was to document conjointly health‐care professionals, patients’ experiences regarding and caregivers their participation in an interdisciplinary pragmatic primary care intervention aimed to improve self‐management of patients with multimorbidity in a French‐Canadian context. By taking an interest in conditions surrounding the intervention, the results of this study may contribute to assessing the process of implementation and to allow better understanding and explain the effects of the intervention. However, given the low number of interviewees in each category, the results of this study may not reflect all participants’ experiences. The possibility of social desirability bias exists and may have influenced participants’ opinions.

Another strength and unique feature about this study was the integration different care models (chronic care model, patient‐centred care, interprofessional care) and approaches (self‐management support, motivational interviewing); and also, the use of an innovative analysis method with triangulation of patients’, family members’ and health‐care providers’ views. Considering these points of view will probably improve the development and implementation of future interventions for the prevention and management of chronic diseases in primary care.

This study has several limitations. Although we tried to recruit participants with both positive and negative experiences of the PCC intervention, it is possible that unsatisfied patients refused to participate in the interviews. In addition, the implementation and evaluation of the PCC intervention were done during the same period (4 months). Despite very positive experiences, it is possible that the intervention did not reach its full potential in that time.

## 5 CONCLUSION

This intervention was useful and rewarding from the health‐care professionals’, patients’ and caregivers’ perspective. However, to ensure the success of this complex multidisciplinary intervention, implementers and managers should anticipate organizational barriers such as availability and time management of relocated health‐care professionals.

## CONFLICT OF INTERESTS

The authors declare that they have no competing interests. All authors listed on the manuscript contributed to the study design, data collection, data analysis and interpretation of the findings, and drafting and reviewing the final manuscript.

## ETHICS APPROVAL AND CONSENT TO PARTICIPATE

The PACE in MM study was approved by the Research Ethics Board of the Integrated University Health and Social Services Centre of Saguenay‐Lac‐St‐Jean (Comité d’éthique de la recherche du Centre intégré universitaire de santé et de services sociaux du Saguenay–Lac‐St‐Jean) (Ethical code 2013‐010). All participants consented to research by signing the approved informed consent form prior to study participation.

## CONSENT FOR PUBLICATION

Not applicable—Information within this manuscript does not contain personal identifiers.

## DATA AVAILABILITY STATEMENT

The data that support the findings of this study are available on request from the corresponding author, [PN]. The data are not publicly available due to their containing information that could compromise the privacy of research participants.
