# Supplementary material for: Patients, caregivers and health‐care professionals’ experience with an interdisciplinary intervention for people with multimorbidity in primary care: A qualitative study
Source: Health Expect. 2020 Feb 8;23(2):318–27. doi: 10.1111/hex.13035 (PMC7104629; doi:10.1111/hex.13035)
Supplement: Supplementary file 1 [file HEX-23-318-s001.docx]

Appendix 1.

Consolidated criteria for reporting qualitative studies (COREQ): 32-item checklist

| **No** | **Item** | **Guide questions/description** |  |
| --- | --- | --- | --- |
| **Domain 1: Research team and reflexivity** | | | |
| Personal Characteristics | | | |
| 1. | Interviewer/facilitator | Which author/s conducted the interview or focus group? | None, but two authors (MCC and MF) supervised closely the work of the professionals conducting the interviews |
| 2. | Credentials | What were the researcher's credentials? *E.g. PhD, MD* | PN: MD, MSc, PhD  CF: MSc  TN: MD, PhD  MS1: RN, PhD  MCC : RN, PhD  FG: RN, PhD  CL: PhD  JBB: PhD  MS2: PhD  MF : MD, MSc, CMFC(F) |
| 3. | Occupation | What was their occupation at the time of the study? | MF : Researcher  MCC : Researcher  BBD : Research assistant  TB : Research coordinator |
| 4. | Gender | Was the researcher male or female? | MF : Male  MCC : Female |
| 5. | Experience and training | What experience or training did the researcher have? | MF : Family physician, MSC, senior researcher  MCC : Registered nurse, PhD and senior researcher |
| Relationship with participants | | | |
| 6. | Relationship established | Was a relationship established prior to study commencement? | No |
| 7. | Participant knowledge of the interviewer | What did the participants know about the researcher? e*.g. personal goals, reasons for doing the research* | The study was presented to all participants before enrollment. All participants signed a consent form.  The goal of the interview was explained to all participants before the interviews. |
| 8. | Interviewer characteristics | What characteristics were reported about the interviewer/facilitator? e.g. *Bias, assumptions, reasons and interests in the research topic* | Two research professionals, a PhD student and two senior researchers (a male and a female) conducted the interviews. All worked with the research team since the beginning of the study. |
| **Domain 2: study design** | | | |
| Theoretical framework | | | |
| 9. | Methodological orientation and Theory | What methodological orientation was stated to underpin the study? *e.g. grounded theory, discourse analysis, ethnography, phenomenology, content analysis* | Qualitative, exploratory, interpretive, descriptive qualitative study (Thorne’s interpretive description approach) embedded in a randomized trial |
| Participant selection | | | |
| 10. | Sampling | How were participants selected? *e.g. purposive, convenience, consecutive, snowball* | Maximum variability was targeted based on age, gender, socio-economic status, practices. |
| 11. | Method of approach | How were participants approached? e*.g. face-to-face, telephone, mail, email* | Patients were already participants in a pragmatic trial. They were contacted by phone offered to extend their participation with this optional qualitative inquiry. The professionals were contacted by phone at their working place. |
| 12. | Sample size | How many participants were in the study? | 30 accepted to participate.   - 9 patients - 5 family members - 16 professionals |
| 13. | Non-participation | How many people refused to participate or dropped out? Reasons? | - Number of patients that refused to participate: unknown (indirect solicitation involving the healthcare professionals). - All (16) professionals contacted accepted to participate. - Number of family members that refused to participate: unknown (indirect solicitation involving the patients) - No participant dropped out |
| Setting | | | |
| 14. | Setting of data collection | Where was the data collected? e*.g. home, clinic, workplace* | Primary health care practices |
| 15. | Presence of non-participants | Was anyone else present besides the participants and researchers? | No |
| 16. | Description of sample | What are the important characteristics of the sample? *e.g. demographic data, date* | Patients were already participants in a pragmatic trial. They all had received the intervention. They were married for the majority with different levels of family income. The majority had 3 or more chronic conditions |
| Data collection | | | |
| 17. | Interview guide | Were questions, prompts, guides provided by the authors? Was it pilot tested? | The interview guides included open questions relating to the healthcare professionals’ expectations and role in the intervention, patients and caregivers’ expectations, barriers and facilitators influencing the efficiency of the intervention, and the impact of the intervention on themselves. Interviews were in French. It was pretested and adjusted accordingly. |
| 18. | Repeat interviews | Were repeat interviews carried out? If yes, how many? | Not with this design |
| 19. | Audio/visual recording | Did the research use audio or visual recording to collect the data? | Al interviews were audio-recorded |
| 20. | Field notes | Were field notes made during and/or after the interview or focus group? | The interviewers took notes during and after the interviews. |
| 21. | Duration | What was the duration of the interviews or focus group? | 23 to 74 minutes |
| 22. | Data saturation | Was data saturation discussed? | Yes |
| 23. | Transcripts returned | Were transcripts returned to participants for comment and/or correction? | No |
| **Domain 3: analysis and findings** | | | |
| Data analysis | | | |
| 24. | Number of data coders | How many data coders coded the data? | Two coders with regular validation with one of the authors (PN). One coder is also co-author (CF). The other coder was an employee of the team at the time of the research. |
| 25. | Description of the coding tree | Did authors provide a description of the coding tree? | No |
| 26. | Derivation of themes | Were themes identified in advance or derived from the data? | The first codes were based on interview guide. Many other codes derived from the data following an inductive approach combined with thematic analysis |
| 27. | Software | What software, if applicable, was used to manage the data? | NVivo 11 |
| 28. | Participant checking | Did participants provide feedback on the findings? | Not indicated (ref Sally Thorne, Interpretative Description: Qualitative Research for Applied Practice. 2016, Taylor and Francis) |
| Reporting | | | |
| 29. | Quotations presented | Were participant quotations presented to illustrate the themes / findings? Was each quotation identified? e*.g. participant number* | Yes. All quotes have been translated to English by a Professional Translator |
| 30. | Data and findings consistent | Was there consistency between the data presented and the findings? | All findings are based on the collected and analyzed data. Major findings are consistent and contrasted. |
| 31. | Clarity of major themes | Were major themes clearly presented in the findings? | Yes. Major themes are described in the manuscript. |
| 32. | Clarity of minor themes | Is there a description of diverse cases or discussion of minor themes? | Yes. Some minor themes related to challenges faced by participants are described. |

Appendix 2: TiDIER CHECKLIST

| **Item** |  |
| --- | --- |
| Provide the name or a phrase that describes the intervention | “…four-month, pragmatic, interdisciplinary intervention for the prevention and management of chronic diseases aimed at supporting self-management of patients with multimorbidity in primary care”  See section Intervention page 3 |
| Describe any rationale, theory or goal of the elements essential to the intervention | See protocol: Stewart M, Fortin M; Patient-Centred Innovations for Persons with Multimorbidity Team*. Patient-Centred Innovations for Persons with Multimorbidity: funded evaluation protocol. CMAJ Open. 2017;5(2): E365–E372. doi:10.9778/cmajo.20160097 |
| Materials: describe any physical or informational materials used in the intervention, including those provided to participants or used in intervention delivery or in training of the intervention providers | Added information:  Each healthcare professional was given a training handbook containing informations about each intervention components.  No specific material was used during interventions. Healthcare professionals used their own material. |
| Procedures: Describe each of the procedures, activities and /or processes used in the intervention, including any enabling or support activities | Section Intervention page 3.  “Patients were assessed for eligibility by family physicians or registered nurses. Each eligible patient was provided with one-hour initial assessment by a primary care nurse to create an intervention plan focused on their needs and according to their objectives and; to direct patients to other healthcare professionals (nutritionists, kinesiologists or the respiratory therapist) according to their intervention plan. Each patient’s intervention had to be based on the educational and coaching content of the training (patient-centered care approach for patients with multimorbidity, self-management support, and motivational interviewing). Interdisciplinary meetings between family physicians, nurses and other health professionals were to be held to discuss cases and harmonize the intervention plan.” |
| For each category of intervention provider, describe their expertise, background, and any specific training given. | Practice Nurse; Nutritionists; Kinesiologists; Respiratory therapists.  Training was given to all healthcare profressionals who worked with chronic diseases patients. This involved being trained on patient-centered care approach for patients with multimorbidity, self-management support, and interprofessional collaboration and motivational interviewing. Their training lasted in average 7.8 hours. |
| Describe the modes of delivery (such as face-to-face or by some other mechanism, such as internet or telephone) of the intervention, and whether it was provided individually or in a group | Interventions were provided individually and face-to-face. |
| Describe the type(s) of location(s) where the intervention occurred, including any necessary infrastructure or relevant features | Recruitment and intervention was undertake in the patient’s FMGs. |
| Describe the number of times the intervention was delivered and over what period of time including the number of sessions, their schedule and their duration, intensity or dose. | Once recruited, patients had an initial assessment with the nurse during approximately one hour. Based on patients’ centered care, no specific number of sessions was planned. Patients could see healthcare professionals as much (or as little) as they wanted during a 4-month period. In average, patients had 2.6 hours of interventions throughout the 4-month period. |
| If the intervention was planned to be personalised, titrated or adapted, then describe what, why, when and how. | See above. |
| If the intervention was modified during the course of the study, describe the changes (what, why, when and how) | N/A |
| Planned: if intervention adherence or fidelity was assessed, describe how and by whom, and if any strategies were used to maintain or improve fidelity, describe them. | Intervention adherence was assessed after intervention completion by the research team. Basically, they focussed on measuring if content of the intervention has been done as planned and how much they have been delivered to participants. |
| Actual: if intervention adherence or fidelity was assessed, describe the extent to which the intervention was delivered as planned. | Intervention adherence will be discussed in another paper that is now in the publication process. |

Appendix 3. Intervention Logic Model

| **Core inputs** | **Activities or program’s component** | **Reached Population** | **Outcomes** | | |
| --- | --- | --- | --- | --- | --- |
|  |  |  | **Short-term** | **Medium-term** | **Long-term** |
| At organisational level | | | | | |
| Establishment of the interdisciplinary team in FMG (component 1) | 1. Inventory of health professionals’ needs for each FMG. 2. Check-up for health professionals’ availability for relocation in FMG. 3. Integration of health professionals in FMG. | Decision-makers;  FMG managers and doctors;  Health professionals | Increased interdisciplinary teamwork (sharing of patient-related information among health professionals).  Reduced patient’s need to tell all his information again and again. | Increased primary care utilisation for patients with chronic conditions. | Reduced inappropriate health care resources utilisation (e.g. emergency department) and costs.  Fostered interprofessional collaboration. |
| At health professionals level | | | | | |
| Health professionals’ coordination and training (component 2 and 3) | 1. Provide training on self-management support, patient-centered care, interprofessional collaboration and motivational approach. 2. Support the community of practice between nurses and FMG’s coordinator. | FMG’s health professionals | Increased knowledge to adopt interventions’ component in health professionals practices.  Fostered information translation during implementation between FMG’s and coordinator. | Increased health professionals’ competency to work with patients with chronic disease.  Support health professionals to fully embrace the program’s components and vision. | Increased health professionals’ work-related quality of life.  Support program’s comprehension and adoption by all health professionals. |
| At patients level | | | | | |
| Patients intervention with a focus on multimorbidity and self-management (component 4-5-6) | 1. Recruitment by a general practitioner or nurse. 2. Nursing initial assessment, the establishment of a care plan of patient’s needs and objectives established by share decision, then referencing to health professionals according to the patient’s needs and objectives. 3. Follow up a consultation by the nurse(s) or health professional(s) targeted by the care plan. | Patients with chronic disease | Increased awareness about patient’s diseases and management. | Increased patients’ confidence to manage his day-to-day condition.  Decreased patients and their families’ general stress. | Improved patients’ self-management, health outcomes, quality of life, health care perception and utilization. |

Program’s logic model

FMG: Family medicine group
